# Supplementary material for: Identification of photoperiod-induced specific miRNAs in the adrenal glands of Sunite sheep (Ovis aries)
Source: Front Vet Sci. 2022 Jul 22;9:888207. doi: 10.3389/fvets.2022.888207 (PMC9354845; doi:10.3389/fvets.2022.888207)
Supplement: Supplementary file 1 [file Data_Sheet_1.ZIP › Supplementary file/miRDeep2 file.zip!/miRDeep2 file/Know_Structure/SPLP42A2/oar-mir-412.pdf]

miRBase precursor : oar-mir-412
Total read count : 468
oar-miR-412-3p read count : 456
oar-miR-412-5p read count : 6
remaining reads : 6

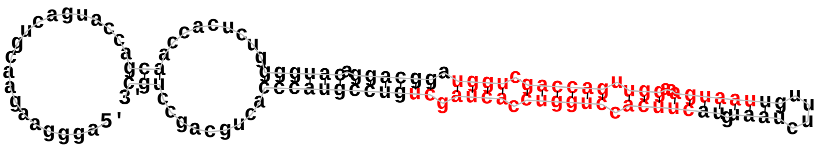

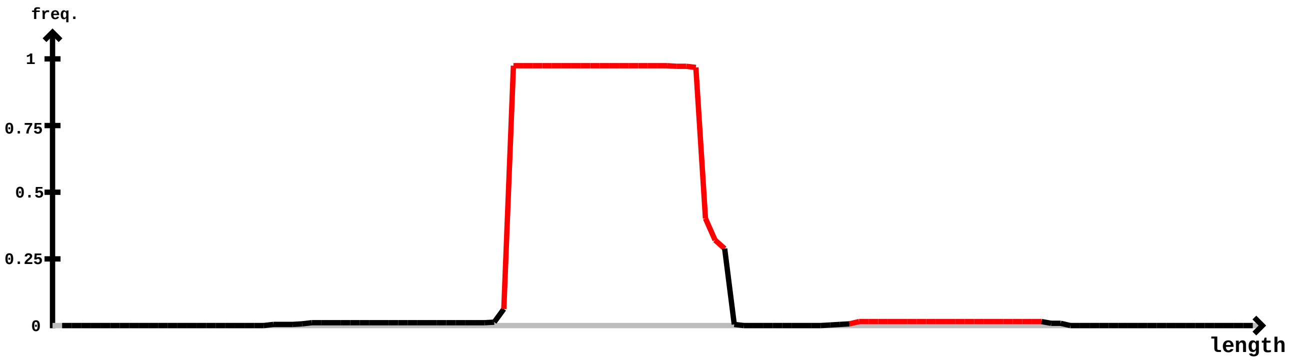


```
                                                                                  oar-miR-412-5p
                                             oar-miR-412-3p
5'- agggaagaacgucaguaccagcaaccacucugggguacaggacggauggucgaccaguuggaaaguaauuguuucuaauguacuucaccugguccacuagcuguccguacccacugcagccugc -3'  exp
    ..........(.((((..(((........)))((((((.((((((.((((.((((((.(((..((((((((....)))).))))))).))))))).)))).))))))))))))))))).......  reads  mm
    .....................caaccacucugggguacaggacgga..............................................................................  2      0
    ........................ccacucugggguacaggacgga..............................................................................  1      0
    .........................cacucugggguacaggacgga..............................................................................  2      0
    ............................................gauggucgaccaguuggaaagu..........................................................  1      0
    .............................................auggucgaccaguuggaaag...........................................................  1      0
    .............................................auggucgaccaguuggaaagu..........................................................  20     0
    .............................................auggucgaccaguuggaaagua.........................................................  1      0
    .............................................auggucgaccaguuggaaaguaau.......................................................  1      0
    ..............................................uggucgaccaguuggaa.............................................................  1      0
    ..............................................uggucgaccaguuggaaag...........................................................  1      0
    ..............................................uggucgaccaguuggaaagu..........................................................  244    0
    ..............................................uggucgaccaguuggaaagua.........................................................  37     0
    ..............................................uggucgaccaguuggaaaguaa........................................................  15     0
    ..............................................uggucgaccaguuggaaaguaau.......................................................  132    0
    ..............................................uggucgaccaguuggaaaguaauu......................................................  2      0
    ...............................................................................guacuucaccugguccacuagcu......................  1      0
    ................................................................................uacuucaccugguccacuagcu......................  1      0
    .................................................................................acuucaccugguccacuagcu......................  1      0
    ..................................................................................cuucaccugguccacuagcugu....................  4      0
```
